# Supplementary material for: Organic-to-Aqueous Phase Transfer of Alloyed AgInS2-ZnS Nanocrystals Using Simple Hydrophilic Ligands: Comparison of 11-Mercaptoundecanoic Acid, Dihydrolipoic Acid and Cysteine
Source: Nanomaterials (Basel). 2021 Mar 25;11(4):843. doi: 10.3390/nano11040843 (PMC8066034; doi:10.3390/nano11040843)
Supplement: Supplementary file 1 [file nanomaterials-11-00843-s001.pdf]

Supplementary Materials

# Organic-to-Aqueous Phase Transfer of Alloyed AgInS2-ZnS Nanocrystals Using Simple Hydrophilic Ligands: Comparison of 11-Mercaptoundecanoic Acid, Dihydrolipoic Acid and Cysteine

Patrycja Kowalik,[1,2] Piotr Bujak,[1]* Mateusz Penkala,[3] Adam Pron[1]

[1]*Warsaw University of Technology, Faculty of Chemistry, Noakowskiego 3, 00-664 Warsaw, Poland. E-mail: piotrbujakchem@poczta.onet.pl*

[2]*Faculty of Chemistry, University of Warsaw, Pasteura 1 Str., PL-02-093 Warsaw, Poland*

[3]*Institute of Chemistry, Faculty of Mathematics, Physics and Chemistry, University of Silesia, Szkolna 9, 40-007 Katowice, Poland*

*Preparation of $AgInS_2$-ZnS nanocrystals (R-"Red" and G-"Green")*

In the synthesis of Ag-In-Zn-S nanocrystals and primary ligand exchange procedures earlier described in the literature were followed **[1,2]**. All operations were carried out under a constant dry argon flow. Silver nitrate (0.03 g, 0.17 mmol), indium(III) chloride (0.13 g, 0.59 mmol), zinc stearate (0.40g, 0.63 mmol for **R** or 0.87 g, 1.37 mmol for **G**), and DDT (0.20 g, 1.00 mmol) were mixed with ODE (15 mL) in a three-neck flask. The mixture was heated to 150 °C until a homogenous solution was formed. Then sulfur (0.015 g, 0.47 mmol) dissolved in 1 mL of OLA was quickly injected into the reaction solution. The temperature was increased to 180 °C, and the mixture was kept at this temperature for 60 min. After the mixture was cooled to room temperature, toluene (20 mL) was added, and the reaction mixture was centrifuged - the isolated black precipitate was separated. The supernatant was treated with 30 mL of acetone leading to the precipitation of the desired fraction of nanocrystals. The nanocrystals (**R** or **G**) were separated by centrifugation (7000 rpm, 5 min) and then redispersed in toluene.

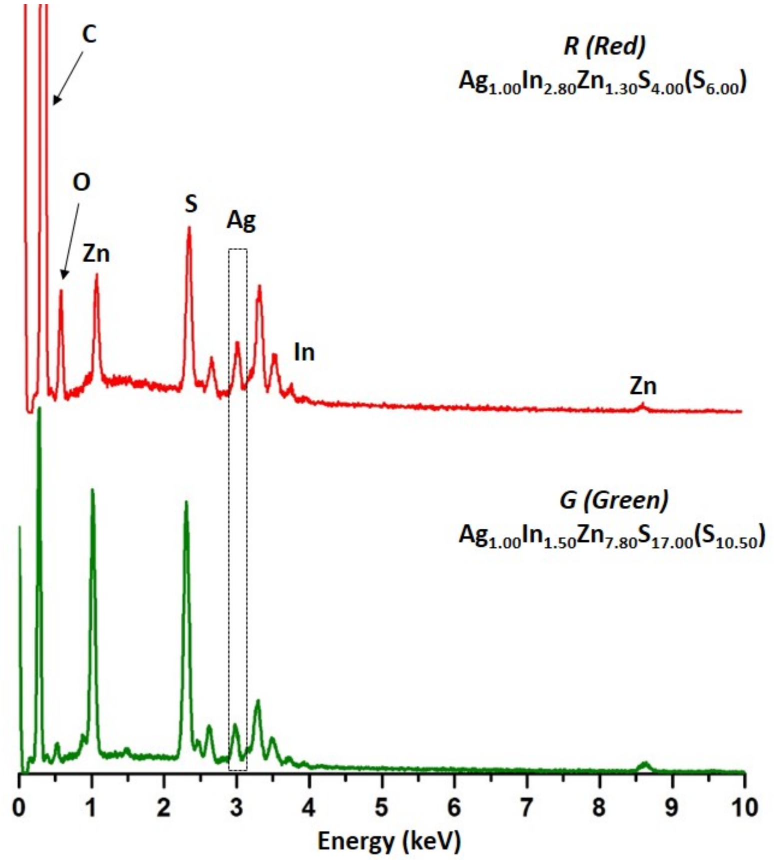


**Figure S1** Energy-dispersive spectra of $Ag_{1.00}In_{2.80}Zn_{1.30}S_{4.00}(S_{6.00})$ (**R**) and $Ag_{1.00}In_{1.50}Zn_{7.80}S_{17.00}(S_{10.50})$ (**G**) nanocrystals.

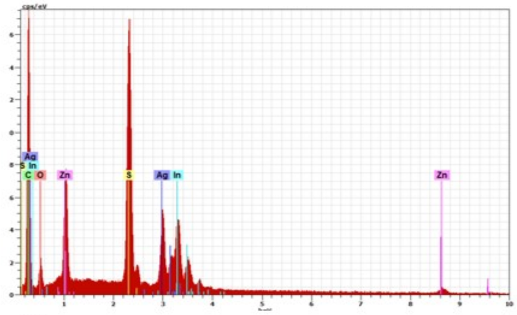


| Element | Series | unn. C [wt.%] | norm. C [wt.%] | Atom. C [at.%] | Error (1 Sigma) [wt.%] |
|---|---|---|---|---|---|
| Carbon | K-series | 42.45 | 42.92 | 74.73 | 5.31 |
| Oxygen | K-series | 6.06 | 6.13 | 8.01 | 1.06 |
| Sulfur | K-series | 13.83 | 13.98 | 9.12 | 0.52 |
| Zinc | L-series | 8.96 | 9.06 | 2.90 | 0.61 |
| Silver | L-series | 13.35 | 13.50 | 2.62 | 0.46 |
| Indium | L-series | 14.25 | 14.41 | 2.62 | 0.47 |
| | Total: | 98.91 | 100.00 | 100.00 | |

***R-MUA***

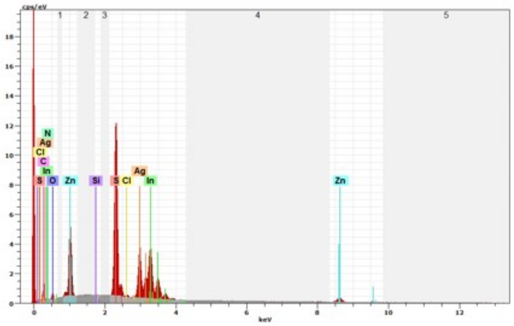


| Element | Series | unn. C [wt.%] | norm. C [wt.%] | Atom. C [at.%] | Error (1 Sigma) [wt.%] |
|---|---|---|---|---|---|
| Sulfur | K-series | 19.56 | 22.97 | 30.05 | 0.72 |
| Indium | L-series | 22.85 | 26.83 | 9.80 | 0.74 |
| Oxygen | K-series | 3.29 | 3.86 | 10.13 | 0.70 |
| Zinc | K-series | 11.55 | 13.56 | 8.70 | 0.50 |
| Carbon | K-series | 7.43 | 8.73 | 30.48 | 1.32 |
| Chlorine | K-series | 0.00 | 0.00 | 0.00 | 0.00 |
| Silver | L-series | 19.98 | 23.46 | 9.12 | 0.67 |
| Nitrogen | K-series | 0.47 | 0.55 | 1.64 | 0.22 |
| Silicon | K-series | 0.04 | 0.05 | 0.07 | 0.03 |
| | Total: | 85.18 | 100.00 | 100.00 | |

***R-DHLA***

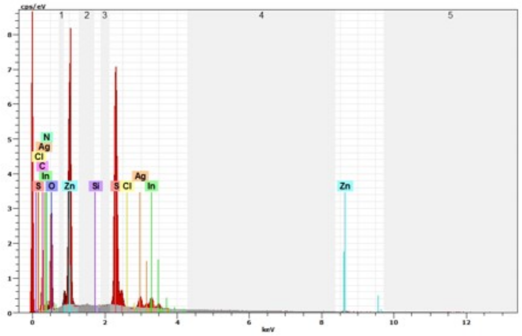


| Element | Series | unn. C [wt.%] | norm. C [wt.%] | Atom. C [at.%] | Error (1 Sigma) [wt.%] |
|---|---|---|---|---|---|
| Sulfur | K-series | 14.07 | 22.51 | 12.45 | 0.53 |
| Indium | L-series | 2.60 | 4.15 | 0.64 | 0.12 |
| Oxygen | K-series | 21.57 | 34.52 | 38.25 | 2.81 |
| Zinc | K-series | 1.12 | 1.79 | 0.48 | 0.10 |
| Carbon | K-series | 16.69 | 26.71 | 39.42 | 2.40 |
| Chlorine | K-series | 0.00 | 0.00 | 0.00 | 0.00 |
| Silver | L-series | 2.44 | 3.90 | 0.64 | 0.11 |
| Nitrogen | K-series | 4.00 | 6.40 | 8.10 | 0.83 |
| Silicon | K-series | 0.01 | 0.02 | 0.01 | 0.03 |
| | Total: | 62.50 | 100.00 | 100.00 | |

***R-Cys***

**Figure S2.** Energy-dispersive spectra of $Ag_{1.00}In_{1.00}Zn_{1.10}S_{3.50}(S_{3.10})$ (**R-MUA**), $Ag_{1.00}In_{1.00}Zn_{1.00}S_{3.30}(S_{3.00})$ (**R-DHLA**) and $Ag_{1.00}In_{1.00}Zn_{0.80}S_{19.40}(S_{2.80})$ (**R-Cys**) nanocrystals.

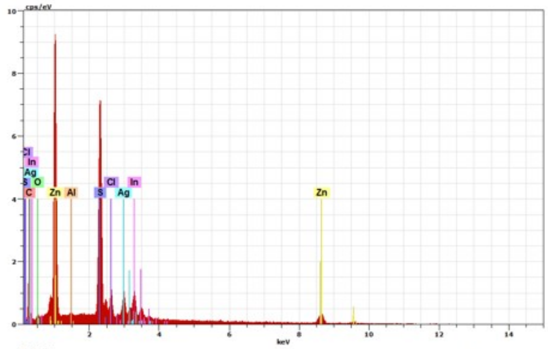

| Element | Series | unn. C [wt.%] | norm. C [wt.%] | Atom. C [at.%] | Error (1 Sigma) [wt.%] |
|---|---|---|---|---|---|
| Carbon | K-series | 42.38 | 50.19 | 76.42 | 6.11 |
| Oxygen | K-series | 6.33 | 7.50 | 8.58 | 1.32 |
| Sulfur | K-series | 11.15 | 13.21 | 7.53 | 0.42 |
| Silver | L-series | 4.00 | 4.73 | 0.80 | 0.16 |
| Indium | L-series | 5.07 | 6.00 | 0.96 | 0.19 |
| Zinc | K-series | 13.53 | 16.02 | 4.48 | 0.54 |
| Aluminium | K-series | 0.09 | 0.11 | 0.07 | 0.03 |
| Chlorine | K-series | 1.90 | 2.25 | 1.16 | 0.09 |
| | Total: | 84.45 | 100.00 | 100.00 | |

*G-MUA*

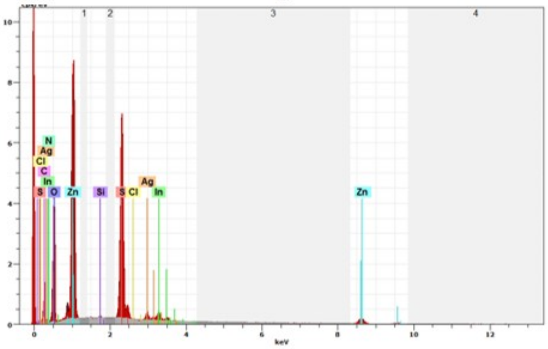

| Element | Series | unn. C [wt.%] | norm. C [wt.%] | Atom. C [at.%] | Error (1 Sigma) [wt.%] |
|---|---|---|---|---|---|
| Sulfur | K-series | 15.64 | 20.73 | 12.86 | 0.58 |
| Indium | L-series | 2.30 | 3.05 | 0.53 | 0.11 |
| Oxygen | K-series | 27.28 | 36.17 | 44.95 | 3.46 |
| Zinc | K-series | 11.19 | 14.84 | 4.51 | 0.47 |
| Carbon | K-series | 15.81 | 20.95 | 34.69 | 2.40 |
| Chlorine | K-series | 0.05 | 0.07 | 0.04 | 0.03 |
| Silver | L-series | 2.07 | 2.75 | 0.51 | 0.10 |
| Nitrogen | K-series | 0.95 | 1.26 | 1.79 | 0.33 |
| Silicon | K-series | 0.13 | 0.17 | 0.12 | 0.03 |
| | Total: | 75.43 | 100.00 | 100.00 | |

*G-DHLA*

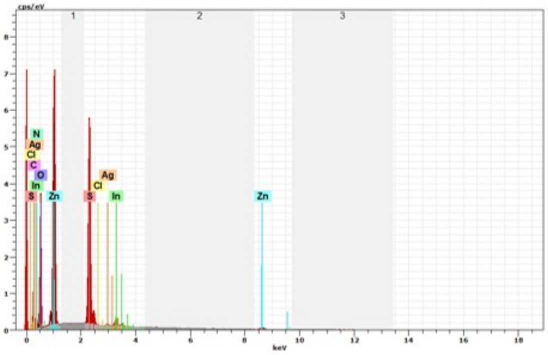

| Element | Series | unn. C [wt.%] | norm. C [wt.%] | Atom. C [at.%] | Error (1 Sigma) [wt.%] |
|---|---|---|---|---|---|
| Sulfur | K-series | 14.36 | 20.93 | 11.36 | 0.54 |
| Indium | L-series | 2.39 | 3.49 | 0.53 | 0.11 |
| Oxygen | K-series | 27.26 | 39.74 | 43.23 | 3.48 |
| Zinc | K-series | 2.81 | 4.09 | 1.09 | 0.17 |
| Carbon | K-series | 16.92 | 24.66 | 35.73 | 2.50 |
| Chlorine | K-series | 0.12 | 0.17 | 0.08 | 0.03 |
| Silver | L-series | 0.39 | 0.57 | 0.09 | 0.05 |
| Nitrogen | K-series | 4.35 | 6.34 | 7.88 | 0.91 |
| | Total: | 68.60 | 100.00 | 100.00 | |

*G-Cys*

**Figure S3.** Energy-dispersive spectra of $Ag_{1.00}In_{1.20}Zn_{5.60}S_{9.40}(S_{7.90})$ (**G-MUA**), $Ag_{1.00}In_{1.00}Zn_{8.80}S_{25.10}(S_{10.80})$ (**G-DHLA**) and $Ag_{1.00}In_{5.80}Zn_{12.10}S_{126.20}(S_{21.30})$ (**G-Cys**) nanocrystals.

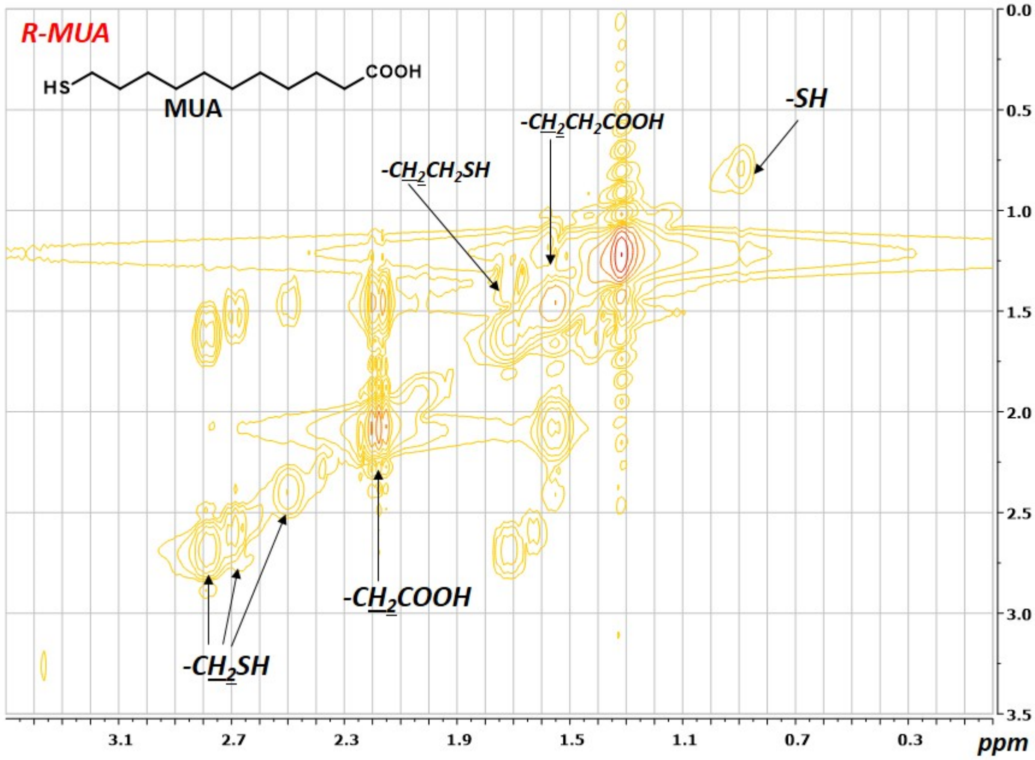


**Figure S4.** $^{1}$H-$^{1}$H COSY spectrum of $D_2O$ dispersion of $AgInS_2$-ZnS nanocrystals capped with MUA: **R-MUA**.

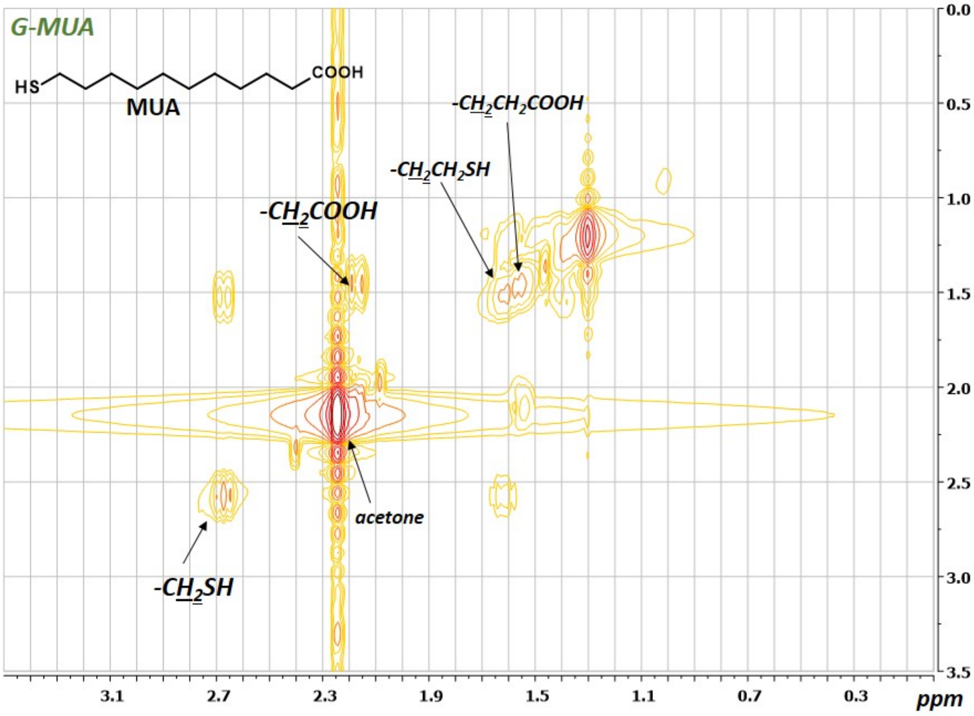


**Figure S5.** $^{1}$H-$^{1}$H COSY spectrum of $D_2O$ dispersion of $AgInS_2$-ZnS nanocrystals capped with MUA: **G-MUA**.

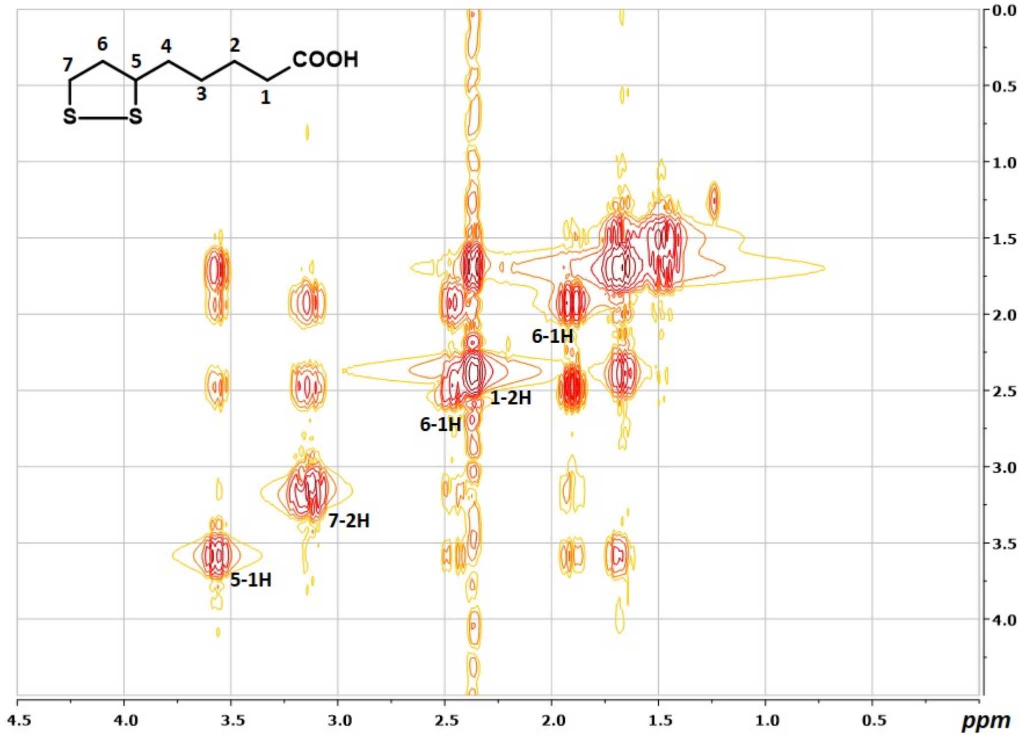


**Figure S6.** $^1$H-$^1$H COSY spectrum of lipoic acid (LA) in $CDCl_3$.

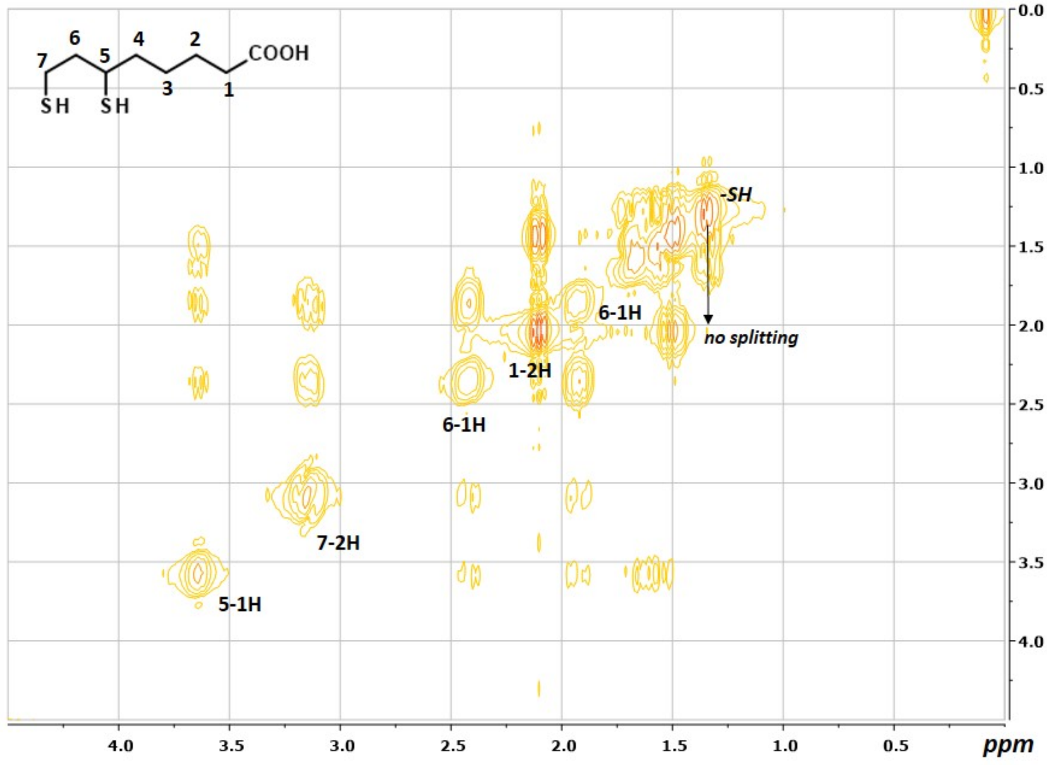


**Figure S7.** $^1$H-$^1$H COSY spectrum of dihydrolipoic acid (DHLA) in $D_2O$.

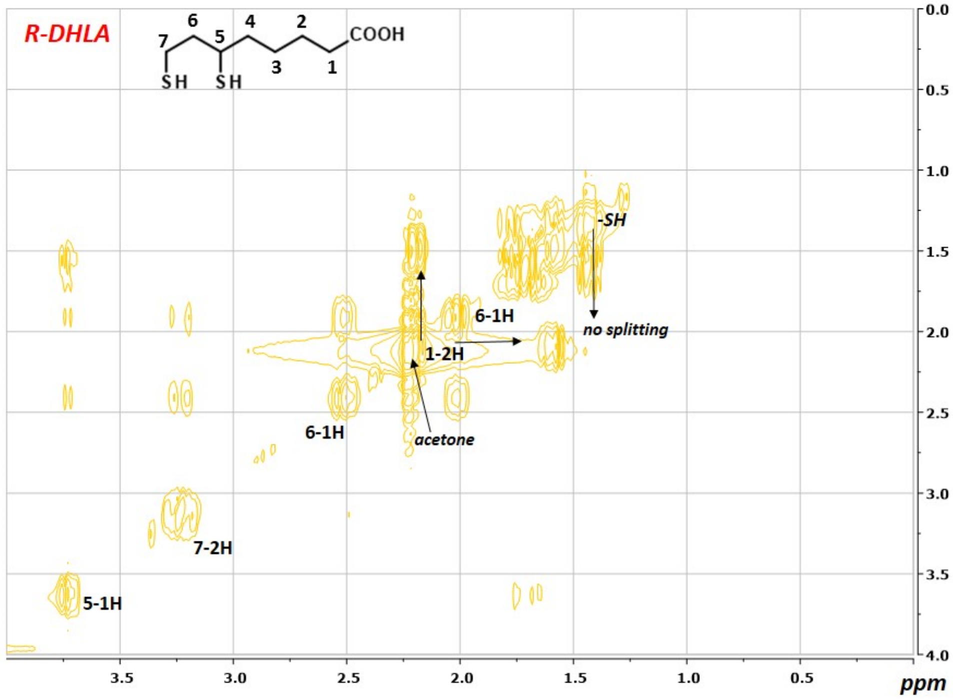


**Figure S8.** $^1$H-$^1$H COSY spectrum of $D_2O$ dispersion of $AgInS_2$-ZnS nanocrystals capped with MUA: **R-DHLA**.

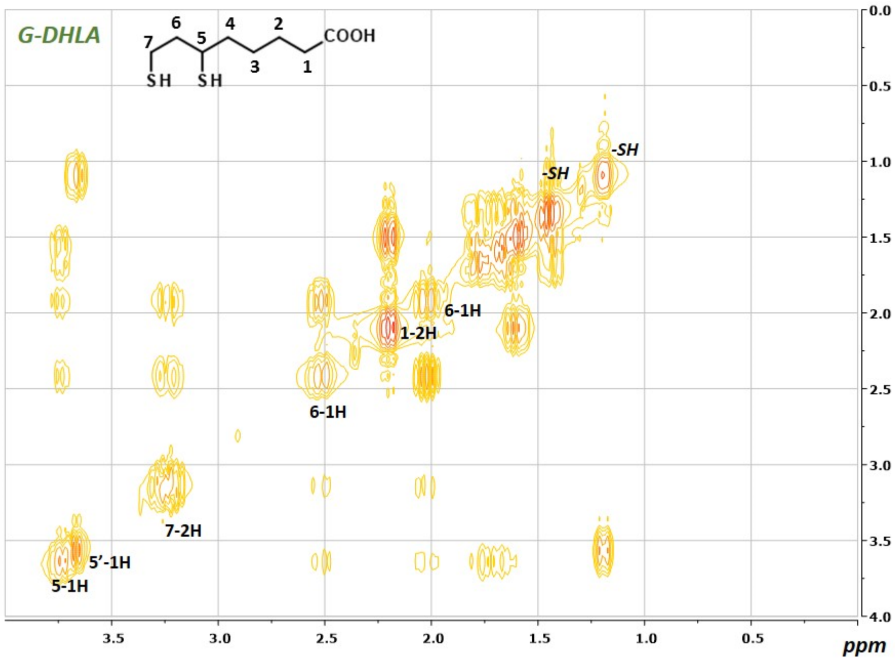


**Figure S9.** $^1$H-$^1$H COSY spectrum of $D_2O$ dispersion of $AgInS_2$-ZnS nanocrystals capped with MUA: **G-DHLA**.

Reference

1. Gabka, G.; Bujak, P.; Giedyk, K.; Ostrowski, A.; Malinowska, K.; Herbich, J.; Golec, B.; Wielgus, I.; Pron. A. A Simple Route to Alloyed Quaternary Nanocrystals Ag-In-Zn-S with Shape and Size Control. *Inorg. Chem.* **2014**, *53*, 5002-5012.

2. Gabka, G.; Bujak, P.; Kotwica, K.; Ostrowski, A.; Lisowski, W.; Sobczak, J.W.; Pron. A. Luminophores of Tunable Colors from Ternary Ag-In-S and Quaternary Ag-In-Zn-S Nanocrystals Covering the Visible to Near-Infrared Spectral Range. *Phys. Chem. Chem. Phys.* **2017**, *19*, 1217-1228.
